# Sialomucin CD43 Plays a Deleterious Role in the Development of Experimental Heart Failure Induced by Pressure Overload by Modulating Cardiac Inflammation and Fibrosis

**DOI:** 10.3389/fphys.2021.780854

**Published:** 2021-12-03

**Authors:** Kuljeet Kaur, Francisco E. Velázquez, Marina Anastasiou, Njabulo Ngwenyama, Sasha Smolgovsky, Mark Aronovitz, Pilar Alcaide

**Affiliations:** ^1^The Department of Immunology, Tufts University School of Medicine, Boston, MA, United States; ^2^Department of Internal Medicine, University of Crete Medical School, Crete, Greece; ^3^Tufts Graduate School for Biomedical Sciences Immunology Program, Tufts University School of Medicine, Boston, MA, United States

**Keywords:** heart failure, inflammation, CD43, T cell, CXCL10

## Abstract

Sialomucin CD43 is a transmembrane protein differentially expressed in leukocytes that include innate and adaptive immune cells. Among a variety of cellular processes, CD43 participates in T cell adhesion to vascular endothelial cells and contributes to the progression of experimental autoimmunity. Sequential infiltration of myeloid cells and T cells in the heart is a hallmark of cardiac inflammation and heart failure (HF). Here, we report that CD43−/− mice have improved survival to HF induced by transverse aortic constriction (TAC). This enhanced survival is associated with improved systolic function, decreased cardiac fibrosis, and significantly reduced T cell cardiac infiltration in response to TAC compared to control wild-type (WT) mice. Lack of CD43 did not alter the number of myeloid cells in the heart, but resulted in decreased cardiac CXCL10 expression, a chemoattractant for T cells, and in a monocyte shift to anti-inflammatory macrophages *in vitro*. Collectively, these findings unveil a novel role for CD43 in adverse cardiac remodeling in pressure overload induced HF through modulation of cardiac T cell inflammation.

## Introduction

Heart failure (HF) is a complex syndrome and the leading cause of hospitalization and mortality worldwide (Virani et al., 2021). Inflammation is one of the hallmarks of HF, and involves a coordinated response between immune cells, the vascular endothelium, and resident cardiac cells that participate in pathological cardiac remodeling, such as cardiac fibroblasts and cardiomyocytes, that are associated with HF progression (Blanton et al., 2019; Carrillo-Salinas et al., 2019; Hanna and Frangogiannis, 2020; Smolgovsky et al., 2021).

Multiple lines of evidence, including our own work, suggest that T cell immune responses contribute to cardiac inflammation and the progression of HF (Laroumanie et al., 2014; Nevers et al., 2015, 2017; Ngwenyama et al., 2021). Using the transverse aortic constriction (TAC) experimental model of non-ischemic HF, we and others have reported that intracellular adhesion molecule-1 (ICAM-1) and endothelial selectin (E-Sel) are highly expressed in the intramyocardial vessels. Moreover, chemokines such as CCL2 and CXCL10 that predominantly recruit CCR2+ myeloid cells and effector T helper type 1 (Th1) cells, respectively, are also present in the heart along with monocytes and T cells (Salvador et al., 2016; Patel et al., 2018; Ngwenyama et al., 2019). Sialomucin CD43 is a highly glycosylated surface protein expressed primarily only in hematopoietic cells with several functions that include serving as an E-Sel ligand for Th1 cells, Th17 cells, and skin resident T cells, and it also facilitates Th17 cell adhesion and apical migration on ICAM-1 (Fuhlbrigge et al., 2006; Alcaide et al., 2012; Velázquez et al., 2016). Genetic deficiency of CD43 in mice results in protection from experimental autoimmunity mediated by T cells, such as experimental autoimmune encephalomyelitis (EAE; Velázquez et al., 2016, 2019). Spontaneous hypertensive rats have been shown to have an increased number of CD43+ monocytes in circulation (Möller et al., 2015). CD43−/− mice are protected from an abdominal aortic aneurism, and deficiency of CD43 in hematopoietic cells prevents the development of atherosclerosis in LDL-R−/− mice (Meiler et al., 2015). We hypothesized that CD43 contributes to adverse cardiac remodeling and HF through mechanisms that involve cardiac inflammation. We report that CD43−/− mice have increased survival to pressure overload-induced HF through mechanisms that involve decreased cardiac expression of CXCL10, cardiac T cell infiltration, and cardiac fibrosis.

## Materials and Methods

### Mice

C57BL/6J (wild-type; WT) and CD43−/− were bred in the pathogen-free facility at Tufts University, according to the institutional animal care and use committee at Tufts University and the NIH Animal research guidelines.

### Mouse Model of TAC and Non Invasive Echocardiography

Left ventricular pressure overload was induced by constricting the transverse aorta of 8–10-week-old male mice to induce HF using a 27G needle, as previously described (Nevers et al., 2015). Sham-operated mice underwent the same procedure but without aortic constriction. *In vivo* transthoracic echocardiography was performed 4weeks after surgery in isoflurane sedated mice. M-mode and two-dimensional images were obtained from the short-axis view as described before (Nevers et al., 2015; Ngwenyama et al., 2021).

### Histological Analysis

Hematoxylin and eosin (H&E) and picrosirius red staining was performed as previously described (Ngwenyama et al., 2019; Carrillo-Salinas et al., 2020). Cardiomyocyte area was determined in H&E stained left ventricle (LV) sections by measuring the area of 10–20 myocytes per heart using ImageJ (NIH; Bethesda, MD). Percent of leukocyte infiltration was determined using ImageJ. 20X image sections were divided into 30 grids, and each grid with infiltrated leukocytes was counted as a positive score. The number of grids containing infiltrated leukocytes (positive grids) was divided by the total number of grids (30) to generate the leukocyte infiltration score (% leukocyte infiltration=the number of positive grids/30×100). Collagen deposition was quantified using ImageJ software.

For immunohistochemistry, OCT frozen sections were fixed in acetone, washed and incubated with 10% normal goat serum for blocking non-specific binding. LV sections were then incubated with anti-mouse CD4 (1:500 dilution; BioLegend, clone GK1.5) or anti-mouse CD11b (M1/70) all purchased from BioLegend, for 1h at room temperature, followed by secondary goat anti-rat biotinylated antibody (1:1,000; Jackson ImmunoResearch, catalog 111-065-003) as described previously (Ngwenyama et al., 2019, 2021).

### RT-PCR

RNA was isolated from heart or cell lysate, where indicated, using RNeasy kit (Qiagen, Valencia, CA, United States) according to the manufacturer’s instructions. RNA was treated with DNase for 15min at room temperature (Qiagen, Valencia, CA, United States). DNA-free total poly A tail RNA (mRNA, 2.0μg) was first subjected to the synthesis of cDNA using Oligo dT primers using SuperScript III First-Strand Synthesis System (Invitrogen, Carlsbad, CA, United States) according to manufacturer’s instructions. About 20ng of cDNA was amplified with SYBR green PCR mix (Applied Biosystems) using specific primers (Table 1).

**Table 1:** Primer sequences used in this study.

Gene	Forward	Reverse
*Coll Ia1*	GTATGCTTGATCTGTATCTG	CGACTCCTACATCTTCTG
*Coll IIIa1*	CCTTGGTCAGTCCTATGAG	CAGGAGCAGGTGTAGAAG
*MHC7*	TGAGCCTTGGATTCTCAAACGT	AGGTGGCTCCGAGAAAGGAA
*MHC6*	CCTAGCCAACTCCCCGTTCT	GCC AAT GAG TAC CGC GTG A
*CXCL10*	ATGACGGGCCAGTGAGAATG	ATTCTTTTTCATCGTGGCAATGA
*Tnfa*	GCACAGAAAGCATGACCCG	GCCCCCCATCTTTTGGG
*Nos2*	GCCACCAACAATGGCAACA	CGTACCGGATGAGCTGTGAATT
*Arg1*	TTGCGAGACGTAGACCCTGG	CAAAGCTCAGGTGAATCGGC
*GAPDH*	AGGTCGGTGTGAACGGATTTG	TGTAGACCATGTAGTTGAGGTCA

### Flow Cytometry

Flow cytometry was used to analyze the immune cell infiltration in the LV. LV was digested with collagenase type II (0.895mg/ml). Infiltrating cells were stained with monoclonal antibodies (mAbs): FITC-conjugated anti-CD4 (clone GK1.5), PE- and APC-conjugated anti-CD4 (clone RM4-5), PE-conjugated anti-CD45.2 (clone 104), and APC-Cy7- and PerCP-conjugated anti-CD11b (clone M1/70). All antibodies were purchased from BioLegend. Cells were surface stained by incubation with the relevant antibodies diluted in PBS+2% FBS for 20min at 4°C, followed by two washes with PBS+2% FBS. Absolute cell numbers were quantified using Precision Count Beads (BioLegend). The data were acquired on an LSRII (Becton Dickinson) and analyzed using FlowJo software.

### Bone Marrow Monocyte Isolation and Macrophage Differentiation

Wild-type and CD43−/− bone marrow (BM) cells were collected from femur and tibias by flushing them with buffer (PBS with 0.5% BSA and 2mM EDTA) using a 25G needle. BM cells were disaggregated and passed through a 40μm cell strainer. From the resulting BM cell suspension, monocytes were allowed to adhere for 48h. Adhered BM-derived monocytes were incubated for 3days with 1μg/ml LPS (Invivogen, tlrl-3pelps) or 20ng/ml of IL-4 (PeproTech 214-14) for differentiation to type 1 macrophages (M1) or type 2 macrophages (M2), respectively. Inducible nitric oxide synthase (iNOS) and Arginase I (Arg-1) expression was determined by qRT-PCR for identifying the M1 and M2 monocyte populations.

### Statistics

All results are presented as mean±SEM. Multiple group comparisons were performed by two-way ANOVA followed by Newman-Keuls multiple comparisons test. Survival curves were compared by log-rank (Mantel–Cox) test. The difference was considered statistically significant at ^*^p≤0.05; ^**^p≤0.01; and ^***^p≤0.001. All statistical analyses were performed using GraphPad Prism. Data is presented as mean±SEM, *n*=5–8 in each group.

## Results

### CD43−/− Mice Have Increased Survival to TAC and Improved Systolic Function as Compared to WT Mice

**Figure 1:**
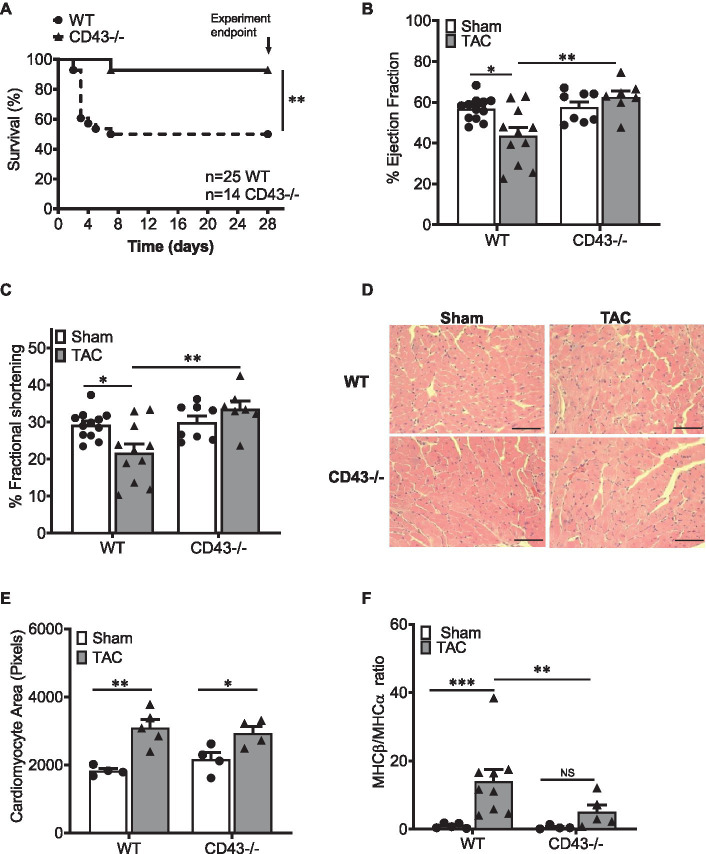
CD43−/− mice are protected from systolic heart failure. **(A)**. Kaplan–Meier survival curves in WT and CD43−/− mice after TAC surgery. Survival curves were compared by log-rank (Mantel–Cox) test *n*=number of animals in survival curve data. Changes in ejection fraction (EF; **B**) and fractional shortening (FS; **C**) in response to TAC in WT and CD43−/− mice as measured by echocardiography. **(D)** Representative images of WT and CD43−/− LV section in Sham and TAC mice. Scale bars, 100μm. **(E)** Quantification of WT and CD43−/− cardiomyocyte area after TAC. **(F)** Quantification of ratio of gene expression for MHCβ and MHCα from heart lysates. ^*^p<0.05; ^**^p<0.01; and ^***^p≤0.001.

To investigate whether CD43 played a role in experimental non-ischemic HF, WT, and CD43−/− age and sex-matched mice underwent TAC and were followed for 4weeks. Strikingly, while 50% of WT mice died during the first week post TAC, only less than 10% CD43−/− mice died, and over 90% of them survived to the experimental endpoint of 28days (Figure 1A). To further understand the reason for such survival, we performed echocardiography and assessed systolic function. As expected, the percent of fractional shortening (FS), and ejection fraction (EF), indices of systolic function were significantly reduced in WT TAC mice compared to WT Sham mice. In contrast, these values remained similar between Sham and TAC CD43−/− mice, and significantly higher in CD43−/− TAC compared to WT TAC mice (Figures 1B,C; Table 2). The LV weight and BNP gene expression was comparable between WT and CD43−/− Sham mice, as well as in response to TAC, where both genotypes experienced a similar increase (Supplementary Figure 1). To further evaluate cardiac hypertrophy, we measured cardiomyocyte area in LV cross-sections from 4weeks TAC or Sham mice. WT mice showed an expected increase in CM area in TAC mice compared to Sham, and, moreover, CD43−/− mice showed a similar increase to WT in response to TAC (Figures 1D,E). Adult to fetal myosin heavy chain (MHCα to MHCβ, respectively) isoform switch, a molecular signature pathological hypertrophy, was also determined as a possible mechanism of preserved systolic function despite a similar increase in LV weight and cardiomyocyte size. WT mice showed an expected increase in the ratio of fetal/adult MHC isoforms (MHCβ/α) in response to TAC as compared to Sham. In contrast, CD43−/− mice exhibited a much lower MHCβ/α isoform ratio in response to TAC as compared to their Sham controls, as well as compared to WT TAC (Figure 1F). Additionally, only WT mice showed increased lung weight, a determinant of pulmonary edema, in response to TAC, whereas CD43−/− mice had comparable lung weights between Sham and TAC mice, which was significantly lower than WT TAC (Supplementary Figure 1B). Taken together, these data demonstrate a deleterious role of CD43 in TAC-induced systolic dysfunction and associated pulmonary edema. Additionally, although WT and CD43−/− mice develop similar LV hypertrophy and cardiomyocyte area, CD43 loss results in decreased MHCβ/α ratio, compared to WT mice in response to TAC.

**Table 2:** Characterization of left ventricular function by echocardiography 4weeks after Sham or transverse aortic constriction (TAC) surgery.

	WT		CD43−/−	
	Sham	TAC	Sham	TAC
LV Vol d	58±4.98	98.33±4.76^*^	65.19±6.90	70.41±13.70^‡^
LV Vol s	25.3±3.15	61.41±5.96^*^	28.85±4.61	27.14±6.20^‡^
LVIDd	3.67±0.13	4.61±0.09	3.85±0.17	3.92±0.30
LVIDs	2.60±0.13	3.76±0.15	2.71±0.18	2.62±0.24
LVPWd	0.89±0.04	1.29±0.12	0.90±0.03	1.31±0.12

LVIDd, left ventricular internal diameter end diastole; LVIDs, left ventricular internal diameter end systole; and LVPWd, left ventricular posterior wall thickness end diastole.

Values are means±SEM.

* *p*<0.05, TAC vs. respective Sham.

‡ *p*<0.05, wild-type (WT) TAC vs. CD43−/− TAC.

### CD43−/− Mice Do Not Develop TAC-Induced Cardiac Fibrosis

**Figure 2:**
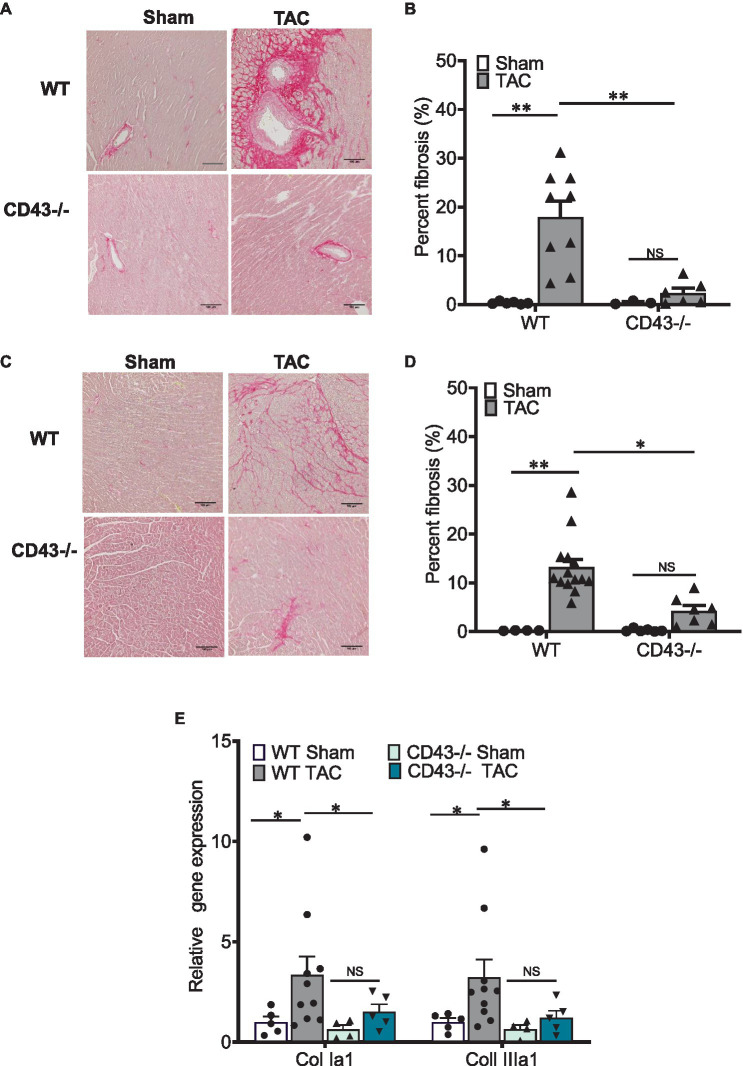
CD43−/− develop less fibrosis in the left ventricle (LV) in response to TAC as compared to WT mice. Representative images of perivascular **(A)** and interstitial **(C)** fibrosis after 4weeks of TAC, as demonstrated by picrosirius red staining. Scale bars, 100μm. Quantification of perivascular **(B)** and interstitial fibrosis **(D)**. **(E)** Quantification of Collagen Ia1 and Collagen IIIa1 gene expression from heart lysates. ^*^p≤0.05; and ^**^p≤0.01.

Another important hallmark of pathological cardiac remodeling is the development of cardiac fibrosis that makes the heart stiff and unable to contract well. We next investigated whether CD43 played a role in cardiac fibrosis and found that unlike WT mice, which develop cardiac perivascular and interstitial fibrosis in response to TAC, CD43−/− mice had significantly reduced perivascular and interstitial fibrosis compared to WT TAC mice (Figures 2A–D). Although CD43−/− TAC mice trended to develop more fibrosis than CD43−/− Sham mice, these differences did not reach statistical significance. qRT-PCR data analysis from heart lysates also showed significantly lower levels of Collagen Ia1 and Collagen IIIa1 gene expression in CD43−/− TAC hearts as compared to the levels seen in WT TAC hearts (Figure 2E). Collectively, these data demonstrate that CD43 contributes to both perivascular and interstitial collagen deposition and fibrosis induced by TAC. The lack of fibrosis in CD43−/− mice may also contribute to the increased survival and improved systolic function observed in CD43−/− TAC mice vs. WT TAC mice.

### CD43 Contributes to Cardiac Immune Cell Infiltration in the LV in Response to TAC

**Figure 3:**
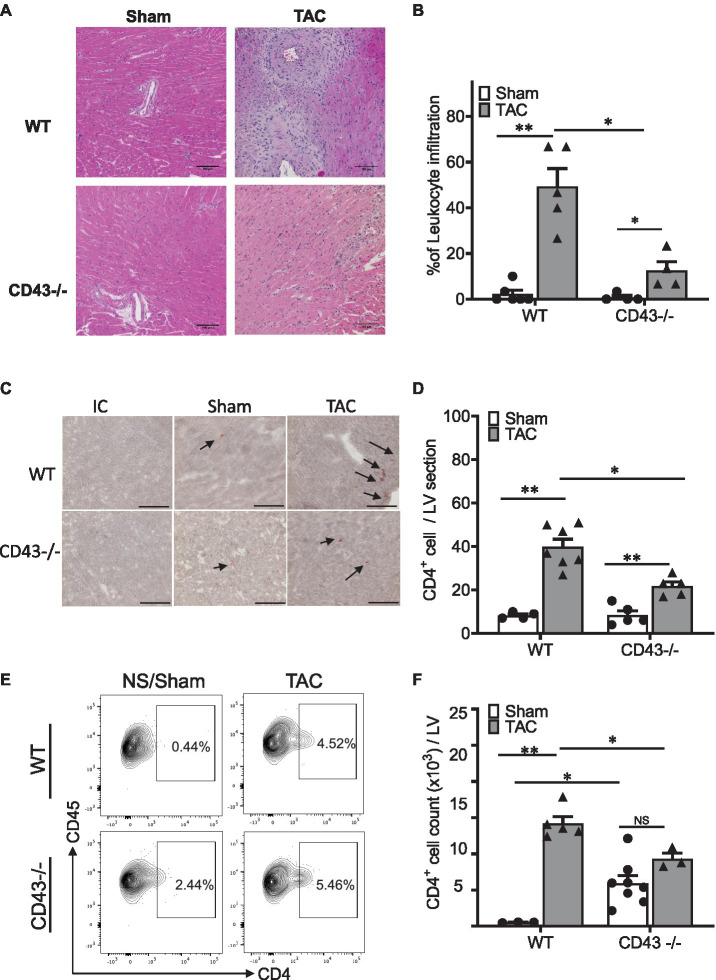
CD43−/− mice exhibit reduced leukocyte and CD4+ T cell infiltration into the LV in response to TAC. **(A)**. Representative hematoxylin and eosin (H&E) staining of WT and CD43−/− heart sections collected 4weeks after TAC and Sham surgery. **(B)** Quantification of leukocyte infiltrates in WT and CD43−/− Sham and TAC LV across the whole heart section. **(C)** Representative CD4 staining images of WT and CD43−/− heart sections from TAC and Sham animals. **(D)** Quantification of CD4+ T cell infiltrates in WT and CD43−/− Sham and TAC LV across the whole heart section. **(E)** Representative flow cytometric panels of CD4+ T cell. **(F)** Quantification of flow cytometric analysis. IC is isotype control; arrows indicate to immune reactivity for CD4 antibody. Scale bars, 100μm. ^*^p≤0.05; and ^**^p≤0.01.

Hematoxylin and eosin staining in Sham and TAC mice revealed that CD43−/− TAC mice had a significantly reduced LV inflammation score that reflects less LV immune cell infiltration than WT TAC mice (Figures 3A,B). Because CD4+ T cells have been reported to induce cardiac fibroblast transformation and fibrosis in WT mice in response to TAC (Nevers et al., 2017), and CD43−/− mice did not develop LV fibrosis in response to pressure overload, we next quantified the presence of CD4+ T cells grossly by analyzing LV cross sections with immunohistochemistry (Figures 3C,D) and with flow cytometry in the digested hearts (Supplementary Figure 2; Figures 3E,F). Using both approaches, we found a higher number of CD4+ LV infiltrated T cells in WT TAC mice compared to Sham mice, as expected. CD43−/− mice, in contrast, had significantly fewer CD4+ LV infiltrated cells in response to pressure overload compared to WT mice (Figure 3). Quantitative flow cytometry of Sham hearts also revealed a slight increase in CD4+ T cells in CD43−/− Sham mice compared to WT Sham mice (Figures 3E,F). These findings demonstrate that CD43 contributes to CD4+ T cell infiltration in the heart in response to cardiac pressure overload and suggest that the reduced fibrosis observed in CD43−/− TAC mice may be as a result of such decreased inflammation mediated by T cells.

### CD43 Does Not Contributes to Myeloid CD11b+ Immune Cell Infiltration in the LV in Response to TAC

**Figure 4:**
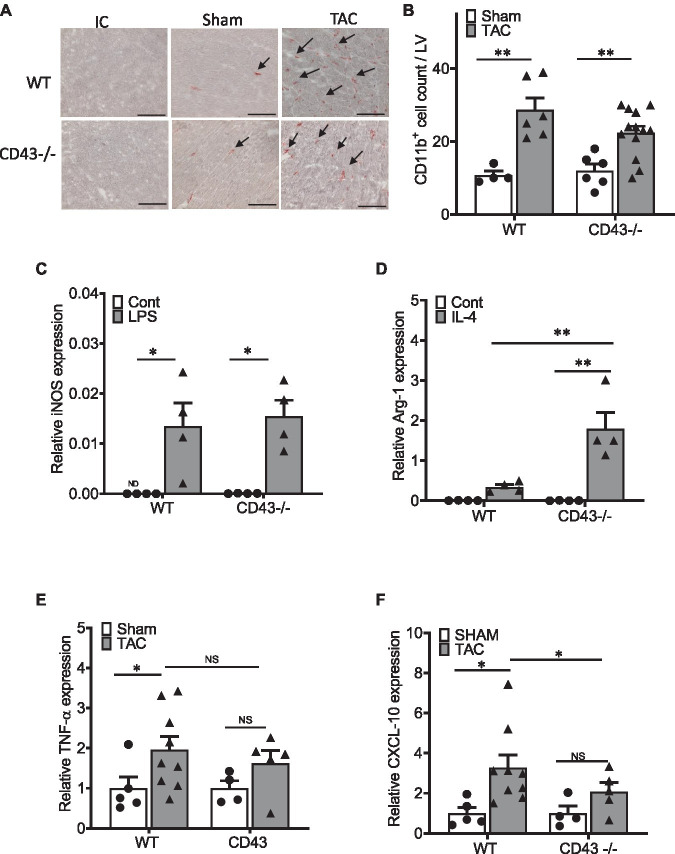
CD43 modulates monocyte polarization toward M2 macrophages *in vitro*, and cardiac CXCL10 expression in response to TAC *in vivo*. **(A)** Representative CD11b staining images of WT and CD43−/− heart sections from TAC and Sham animals. **(B)** Quantification of CD11b infiltrates in WT and CD43−/− Sham and TAC LV across the whole heart section. IC is isotype control; arrows indicate immune reactivity for CD11b antibody. Scale bars, 100μm. **(C,D)** qPCR quantification of inducible nitric oxide synthase (iNOS; **C**) and Arginase I (Arg-1; **D**) in untreated and LPS **(C)** or IL-4 **(D)** treated bone marrow (BM) derived monocytes from WT and CD43−/− mice after 72h of stimulation. **(E,F)** qPCR quantification of TNFα **(E)** and CXCL10 **(F)** of WT and CD43−/− hearts harvested 4weeks after Sham or TAC surgery. ^*^p≤0.05; and ^**^p≤0.01.

Myeloid cells have been reported to precede T cell cardiac infiltration in response to cardiac pressure overload. Because CD43 is also expressed in myeloid cells, we next analyzed the presence of CD11b+ myeloid cells in Sham and TAC hearts from WT and CD43−/− mice. Immunohistochemistry was performed to visualize and quantify infiltrating CD11b+ in LV cross sections (Figures 4A,B). We found that infiltration of CD11b+ cells was similarly increased both in WT and CD43−/− mice in response to TAC. Myeloid cell CD43 has been reported to modulate macrophage function in the context of atherosclerosis. Thus, we hypothesized that monocytes from WT and CD43−/− were phenotypically different. We focused on the monocyte pro-inflammatory phenotype and tested this hypothesis *in vitro* using BM-derived monocytes from CD43−/− and WT mice in *in vitro* conditions that induce polarization toward the type-2 anti-inflammatory macrophages (also known as M2) that express Arg-1, or toward type-1 pro-inflammatory macrophages (also known as M1) expressing iNOS. LPS induced similar transformation of WT and CD43−/− BM-derived monocytes toward M1 macrophages, using iNOS as a readout (Figure 4C), indicating that CD43 does not contribute to pro-inflammatory macrophage transformation *in vitro*. In contrast, IL-4 treatment resulted in increased Arg-1 expression in CD43−/− monocytes compared to WT monocytes, supporting that CD43 prevents the formation of anti-inflammatory M2 macrophages in M2 polarizing conditions *in vitro* (Figure 4D).

The pro-inflammatory cytokine TNF-α, which can be released by pro-inflammatory macrophages, was also similarly increased in the hearts of WT and CD43−/− mice in response to cardiac pressure overload, compared to Sham controls, suggesting that CD43 does not contribute to cardiac TNF-α expression in experimental HF (Figure 4E). A characteristic of pro-inflammatory cardiac infiltrating myeloid cells is the production of the chemokine CXCL10, which is a potent chemoattractant for Th1 cells and is mainly secreted by immune cells (Ngwenyama et al., 2019; Anastasiou et al., 2021). Given the striking decrease of CD4+ T cells in the CD43−/− TAC hearts, we next investigated cardiac CXCL10 expression. We found that cardiac CXCL10 expression was reduced in CD43−/− mice compared to WT mice in response to cardiac pressure overload induced by TAC (Figure 4F). Taken together, these data indicate that CD43 does not contribute to CD11b+ myeloid cell infiltration to the heart after TAC yet it modulates cardiac CXCL10 expression in response to TAC *in vivo*, and contributes to anti-inflammatory M2 macrophage differentiation in *in vitro* polarizing conditions.

## Discussion

In the present study, we report for the first time that CD43−/− mice are protected from pathological cardiac remodeling in response to cardiac pressure overload induced by TAC, through mechanisms that involve modulation of cardiac fibrosis, CXCL10 cardiac gene expression, and T cell mediated inflammation. Consequently, genetic deficiency of CD43 results in improved survival to experimental non-ischemic HF.

Perhaps the most striking finding we report is the preserved systolic function that confers improved survival of CD43−/− mice to experimental HF. One likely explanation is the decreased presence of cardiac T cell infiltration, given that cardiac infiltrated T cells drive cardiac fibrosis in this experimental model of HF in WT mice. The highly glycosylated isoform of CD43 has been reported to function as an E-Sel ligand for cutaneous lymphocyte associated antigen (CLA)+ T cells, a type of pro-inflammatory T cells associated for skin inflammation (Fuhlbrigge et al., 2006). Th17 cells also use CD43 to roll on vascular endothelial cells adhering to E-sel, and to facilitate adhesion on ICAM-1 *in vitro*. Not surprisingly, CD43−/− mice, have decreased T cell infiltration in experimental autoimmunity models that are highly dependent on recruitment of Th17 cells, such as EAE (Velázquez et al., 2016). However, the possibility that CD43 in T cells directly mediates recruitment into the heart in response to cardiac pressure overload is unlikely, mainly because although E-sel and ICAM-1 are expressed in the intramyocardial vessels in TAC hearts (Salvador et al., 2016), there are very few Th17 cells expanded in WT mice in response to TAC, in contrast to a significant expansion of Th1 cells (Laroumanie et al., 2014; Velázquez et al., 2016; Nevers et al., 2017). Furthermore, CD43 alone in Th1 cells is not sufficient to mediate interactions (Alcaide et al., 2012; Velázquez et al., 2016). CD43−/− mice are protected from developing abdominal aortic aneurysm through mechanisms that involve CD43 modulation of IFN-γ secretion by CD8+ T cells (Zhou et al., 2013). Because IFN-γ is profibrotic in TAC, as well as in Angiotensin II-induced hypertension, in which T cells also contribute to cardiac remodeling (Han et al., 2012), it is possible that the observed lack of fibrosis in CD43−/− TAC hearts is a result of this mechanism rather than a direct effect of CD43 in T cell recruitment across the myocardial vessels.

Myeloid cells infiltration into the heart precedes T cell infiltration and the development of cardiac fibrosis and systolic dysfunction (Patel et al., 2018). However, we found that CD43 does not contribute to myeloid cell cardiac infiltration in response to TAC. These results are in line with a study demonstrating that the lack of CD43−/− in leukocytes conferred protection from atherosclerotic plaque development in the pro-atherogenic LDL-R−/− mice, without affecting vascular macrophage infiltration (Meiler et al., 2015). This study highlighted how the lack of CD43 in macrophages impairs cholesterol homeostasis and the formation of the atherosclerotic plaques. In line with an intrinsic function of CD43 in myeloid cells, our *in vitro* data demonstrate that CD43 plays a role in macrophage switching from anti-inflammatory M2 type to pro-inflammatory M1. This distinction needs to be taken carefully to make extrapolations of conclusions *in vivo*, where a more complex setting and interplay of broad spectrum macrophage plasticity takes place (Epelman et al., 2014; Swirski and Nahrendorf, 2018). Nevertheless, our *in vitro* data support a role for myeloid cell CD43 in macrophages that potentially associate with cardiac inflammation *in vivo* (Meiler et al., 2015). Our data demonstrating that CD43−/− mice have significantly decreased cardiac levels of CXCL10, despite the similar numbers of myeloid cells in the heart, indirectly support a less inflammatory role for CD43−/− monocytes as compared to WT. Moreover, these data support a mechanism by which T cells cannot be chemoattracted to the heart, given the key role the CXCL10-CXCR3-ICAM-1 axis plays in T cell recruitment to the heart (Ngwenyama et al., 2019) in response to cardiac pressure overload. In previous studies, we identified myeloid cells, and, to a lesser extent, cardiac fibroblasts, as the main source of CXCL10 in TAC hearts. The present study does not report the cell source of CXCL10 in CD43−/− mice, other than a decrease in cardiac CXCL10 in CD43−/− TAC vs. WT TAC mice. Because CD43 expression is limited to hematopoietic cells, this could also reflect a less pro-inflammatory myeloid phenotype, although this requires further investigation. CD43 has also been reported to contribute to antigen presentation in the immune synapse (Allenspach et al., 2001; Mody et al., 2007). We recently reported cardiac antigen recognition by T cells within the heart (Ngwenyama et al., 2021), and it is possible that the decreased inflammation observed in CD43−/− mice is not only as a result of decreased T cell infiltration due to reduced CXCL10, but also to reduced cardiac antigen engagement. Further studies are needed to confirm this possibility.

Given that CD43 expression is limited to leukocytes, we propose that the inflammatory environment predominantly mediated by T cells contributes to pathological cardiac hypertrophy, although, the specific mechanism involved in hypertrophy remains to be explored in future studies. Other plausible mechanisms may involve decreased pro-fibrotic or pro-hypertrophic factors released by CD43 deficient T cells or macrophages that may impact cardiomyocyte or cardiac fibroblast function. A secretome analysis of the factors released, as recently reported for ischemic cardiac injury (Kuhn et al., 2020), will provide a better insight into the mechanisms playing a protective role in this study.

Our data also highlights a role for CD43 in MHC gene expression. Both CD43−/− and WT cardiac myocytes similarly increased in size in response to TAC, however, only when CD43 is present, the switch from the adult to fetal MHC characteristic of pathological HF occurs. Some limitations besides this one should be additionally acknowledged. Our study is limited to one experimental model of heart failure that is not ischemic in nature. Given the role of myeloid and T cells in cardiac repair post-ischemia, it will be interesting to explore the role of CD43 in this setting. Although in our lab, we do not observe cell death as a major hallmark of cardiac pathology at 4-week post TAC, we cannot discard that later on, when mice develop chronic-end-stage non ischemic heart failure and myocyte death is apparent, CD43 is involved in this process. Similarly, the observed changes in MHC expression in CD43−/− mice will be interesting to investigate in experimental models of physiological hypertrophy, characterized by a distinct gene program in myocytes that may be altered by CD43, or, alternatively, in time points post TAC in which myocytes sense pressure overload and myeloid and T cells start to infiltrate the heart. Because CD43 is exclusively expressed in leukocytes and not in myocytes, a time point in which these co-exist in the heart such as the one studied here, is informative but not complete. Our conclusions are based in loss of function of CD43, and do not include gain of function studies that would require CD43 overexpression in primary mouse monocytes and its outcome in M1 differentiation. Lastly, we only used male mice in this study, and we cannot conclude whether this detrimental role of CD43 in TAC is sex specific.

Despite these limitations, this study describes a comprehensive characterization on the novel role of CD43 in adverse cardiac remodeling using a pre-clinical model of HF. Our data contribute to a better understanding of new mechanisms of cardiac inflammation in HF, and highlights the importance of understanding the impact of cardiac inflammation in adverse cardiac remodeling and cardiac physiology and function, while shedding new light into the complex roles for sialomucin CD43 in cardiovascular and immune cell biology.

## Data Availability Statement

The original contributions presented in the study are included in the article/Supplementary Material, further inquiries can be directed to the corresponding author.

## Ethics Statement

The animal study was reviewed and approved by Tufts University IACUC.

## Author Contributions

KK and FV performed all experiments, analyzed the data, and wrote the manuscript. MA performed some of the flow cytometry studies and the survival curve. NN assisted with *in vivo* experiments. SS performed and analyzed echocardiography and flow cytometry data. MA performed transverse aortic constriction surgeries. PA designed the experiments and supervised the results and manuscript writing. All authors contributed to the article and approved the submitted version.

## Funding

This work was supported by National Institutes of Health Grants HL123658 (to PA) and HL123658-01S1 (to FV).

## Conflict of Interest

The authors declare that the research was conducted in the absence of any commercial or financial relationships that could be construed as a potential conflict of interest.

## Publisher’s Note

All claims expressed in this article are solely those of the authors and do not necessarily represent those of their affiliated organizations, or those of the publisher, the editors and the reviewers. Any product that may be evaluated in this article, or claim that may be made by its manufacturer, is not guaranteed or endorsed by the publisher.
