# Supplementary material for: Sialomucin CD43 Plays a Deleterious Role in the Development of Experimental Heart Failure Induced by Pressure Overload by Modulating Cardiac Inflammation and Fibrosis
Source: Front Physiol. 2021 Dec 3;12:780854. doi: 10.3389/fphys.2021.780854 (PMC8678270; doi:10.3389/fphys.2021.780854)
Supplement: Supplementary file 1 [file Data_Sheet_1.pdf]

Figure **1s**

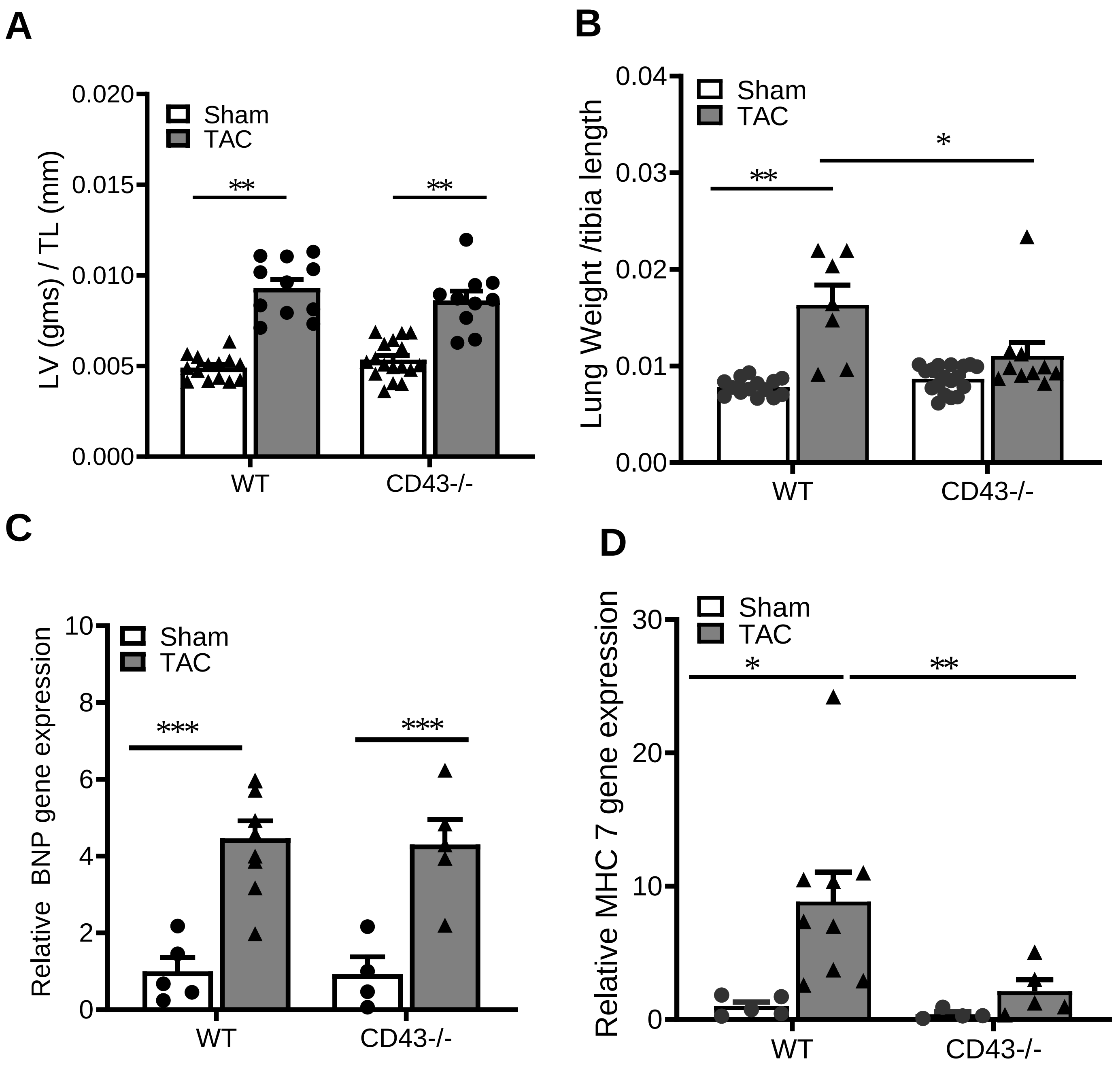

Figure 1S: CD43-/- mice present similar levels of cardiac hypertrophy, do not develop pulmonary edema and have significantly reduced expression of MHCβ expression compared to WT mice in response to TAC.

A). Changes in LV weight normalized to tibia length in response to TAC in WT and CD43-/- mice. B) Changes in Lung Weight in response to TAC in WT and CD43-/- mice qPCR Quantification of gene expression of BNP (C) and MHCβ (D) from heart lysates 4 weeks after Sham or TAC surgery. Data is presented as mean±SEM, n=4–6 in each group. *p<0.05, **p<0.01

Figure **2s**

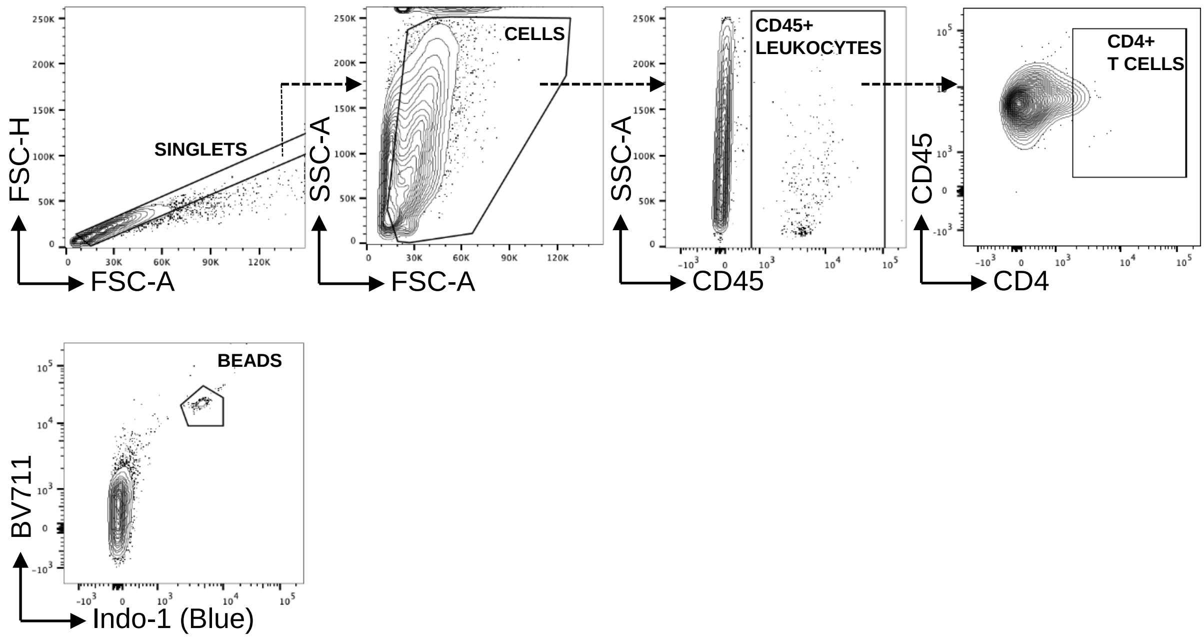

Figure 2S: Representative gating strategy for detection of CD4+ cells in heart. The bottom left panel gate represents the counting beads, which are used for quantification of cell number.

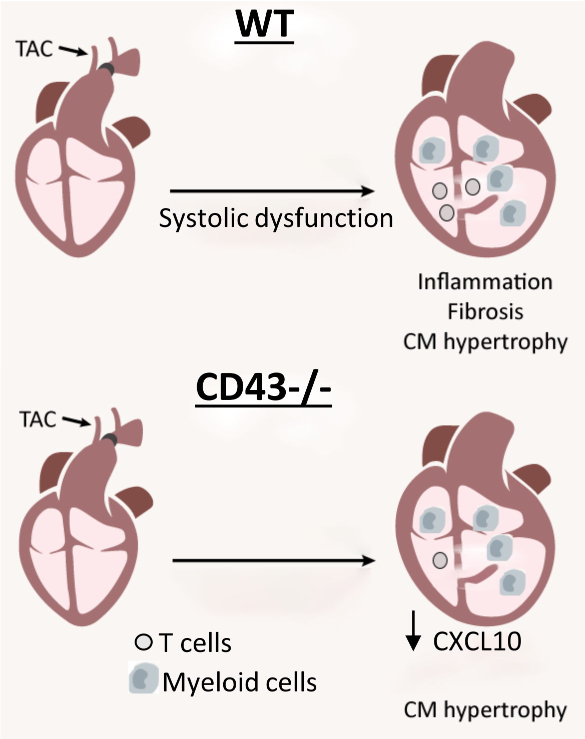

Graphical abstract: CD43 modulates cardiac inflammation and systolic dysfunction in experimental heart failure
